# Psychological distress among patients of an orthopaedic outpatient clinic: a study from a low-income country

**DOI:** 10.1186/1744-859X-9-9

**Published:** 2010-02-15

**Authors:** Nusrat Husain, Syed M Humail, Imran B Chaudhry, Raza Rahman, Holly Robinson, Francis Creed

**Affiliations:** 1 University of Manchester, Manchester, UK; 2 Lancashire Care NHS Foundation Trust, Preston, UK; 3 Dow University of Health Sciences, Karachi, Pakistan

## Abstract

**Background:**

Depression is common among general trauma patients and is associated with a poor outcome. We evaluated the relationship of psychological distress to physical injury, musculoskeletal complaints, and social factors in a low-income country.

**Methods:**

We administered the Self-Rating Questionnaire (SRQ), the Oslo social support questionnaire, and the Brief Disability Questionnaire (BDQ).

**Results:**

An SRQ score of 9 or more, which indicates probable depressive disorder, occurred in 45.6% of men and 76.1% of women. A high SRQ score was associated with female sex, little or no education, low income and little social support. Even after these were controlled for there was a significantly higher SRQ score in patients with arthritis, backache/prolapsed disc, major fracture and other bone pathology.

**Conclusions:**

Depressive disorder appears to be very common in orthopaedic outpatients in Pakistan; both social circumstances and nature of bone pathology are associated with such depression.

## Background

An estimated 340 million people across the world have major depressive disorder at any given time [1]. This disorder is projected to be the leading cause of disability in the developing world by 2020 and leads to greatly increased healthcare costs even after excluding direct mental healthcare expenses [2]. Depressed people are more likely than others to seek medical treatment in the general health sector where underdiagnosis and undertreatment of depression is common. The combination of a physical illness and depressive disorder is a particularly potent cause of disability [3]. Musculoskeletal complaints are the most common self-reported problems in the community [4]. Chronic pain is also one of the most common presentations for healthcare and it is reported that such patients are five times more likely to utilise health than the general population [5,6]. Both regional (for example, in the limbs) and widespread pain are increased in people of Pakistani, Indian and Bangladeshi origin relative to the local white European population in the UK [7,8].

In high-income countries increased rates of depression have been reported in general trauma patients [9-11] and in one US orthopaedic population the rate of depression was 45% [12]. Depression does not appear to be associated with severity of the physical illness or injury but it tends to be persistent and predicts a poor outcome [11,13-15]. To our knowledge there are no reports from any developing country evaluating the relationship of psychological distress, physical injury and musculoskeletal complaints. Since depression is common in Pakistan [16,17], including among attendees at a medical outpatient clinic [18] one might expect a high rate of depression associated with physical injury and musculoskeletal issues presenting at orthopaedic clinics.

In this study we aimed to study the prevalence of depressive symptoms in patients presenting to the orthopaedic outpatient clinic in a large teaching hospital in Karachi, Pakistan. We hypothesised that the prevalence of depression would be greater in the patients with medically unexplained musculoskeletal symptoms compared to those with organic disease in patients attending an orthopaedic clinic in Pakistan even after adjusting for the sociodemographic features known to be associated with depression.

## Methods

### Study setting

The study was conducted over 3 months (January to March 2007) at an orthopaedic outpatient clinic at Civil Hospital in Karachi, Pakistan. Karachi is the largest city in Pakistan; the literacy rate is over 60% compared to 40% for the rest of the country. Poverty is common. The Civil Hospital is one of the largest government hospitals in Pakistan and is a university hospital attached to the Dow Medical College. It has about 1,700 beds and over 35,000 patients are admitted per year. The hospital maintains 37 wards, 11 operation theatres, 15 outpatient departments (OPDs) and 30 other departments including CT scan, mortuary and x ray. The OPD treats over 600,000 patients per year, at a flat rate of 1 rupee per person (€0.008) and approximately 170,000 patients are seen annually in the emergency department.

In Pakistan the system of family practice is not well developed. The majority of the patients attend their local area doctor, a homeopathist or a traditional healer. Patients who do not improve, or who are not able to afford further private treatment, refer themselves to one of the government hospitals. It is known that approximately 25% of patients reaching the psychiatric service do so after being seen in the general hospital [19]. The patients first present to the outpatient clerk who will direct all patients with musculoskeletal complaints to the orthopaedic outpatient department. The Research Ethics Committee of the Pakistan Institute of Learning and Living approved the study.

### Sample

The study was conducted in the orthopaedic outpatient clinic for 1 day a week over 12 consecutive weeks. All patients aged 16 to 65 years who came to consult the doctor were invited to complete the Self-Rating Questionnaire (SRQ) [20] in Urdu (the national language of Pakistan), while waiting for the doctor. The approach to the patients was made by one of six research assistants, three male and three female, (three doctors and three nurses) so that each patient was interviewed by a research assistant of the same sex. The explanation indicated that the doctor would like the patient to complete the questionnaires as part of a survey of general health being carried out on all patients seeing the doctor that day.

After explaining the study in full and obtaining written informed consent, the research assistants administered the Urdu translation of the screening instruments: the SRQ to detect minor psychiatric disorders, Oslo scale for social support and Brief Disability Questionnaire (BDQ) for disability. The research assistants attempted to ensure that the SRQ was completed before the patient left the building. The research assistants also recorded the patient's name, sex, marital status, employment, and the patients' stated reason of consultation with the doctor. The research assistants were trained by the author (NH) in the use of the SRQ and other measures. The surgeon documented the diagnosis and whether the presenting issue was medically explained or not.

### Measures

The SRQ was developed by the World Health Organization and has been widely used in studies in Pakistan [21] and in other developing countries [20]. It is a self-administered questionnaire, which consists of 20 questions with yes/no answers exploring symptoms of depression, anxiety, and somatic manifestations of distress. It has been validated for use in many developing countries [20] and has good psychometric properties. In this study the research assistants read out the SRQ questions to all patients whether they were literate or not in order to maintain consistency. In a rural setting in Pakistan we found that a cut-off of 8/9 had a sensitivity of 80% and a specificity of 85.4% for detecting depressive disorder [16,21]. ROC analysis was used to estimate the optimal threshold score for SRQ.

### Social support

Social support was measured using the Oslo-3 scale which includes three questions identifying the number of people who are very close and offer support when needed, the degree of concern shown by others to the individual and the degree of support received from neighbours when necessary. This has been used in several studies including Pakistanis living in Norway and in Pakistan [22]. The three individual items (each scored 0 to 4) are used in this study with a high score indicating greater social support.

### Disability

Disability was assessed using the crossculturally validated BDQ, which includes six items from the Short-Form 36 item (SF-36) questionnaire [23], asking participants whether they had been limited in various everyday activities during the last month and four questions concerning daily functioning [5]. This has been used in several international studies including Pakistan [21,22]. A total score is used in this study with a high score indicating greater disability.

### Diagnosis

An organic diagnosis was defined as a physical condition where an objective finding was present, which was considered by the surgeon as a likely explanation for the presenting condition. Most of those listed in Table 1 are self-explanatory. 'Other bone pathology' includes ostoemyelitis, cysts, tumours, osteoporosis and TB. Where there was no evidence of an organic disease that could explain the presenting symptoms, the symptom was described as a medically unexplained symptom; these complaints were usually of the chest, ribs, body, muscle, joint or bone pains.

**Table 1:** Mean (standard deviation) of Self-Rating Questionnaire (SRQ) scores by orthopaedic diagnosis

	Overall		Men		Women	
	
	Mean (SD)	N	Mean (SD)	N	Mean (SD)	N
Major fracture	10.14 (5.5)^a^	243	9.07 (5.1)	146	11.74 (5.8)	97
Small fracture	9.74 (6.3)	77	7.25 (5.8)	39	12.29 (5.9)	38
Other bone pathology	12.50 (5.2)^b^	78	9.62 (5.8)	24	13.78 (4.4)	54
Sprain and others	8.12 (6.2)	24	5.62 (4.6)	13	11.09 (6.7)	11
Arthritis	12.03 (5.7)^c^	238	8.02 (5.1)	49	13.07 (5.4)	189
PID/backache	12.43 (4.7)	65	10.70 (4.9)	27	13.65 (4.1)	38
Dislocation	8.2 (7.3)	10	5.00 (6.8)	5	11.4 (6.9)	5
Frozen joint	10.41 (5.9)	17	7.67 (5.0)	6	11.9 (5.9)	11
Wound	9.68 (5.8)	114	7.60 (4.9)	55	11.62 (5.9)	59
Other	8.38 (5.2)	21	7.62 (4.4)	8	8.85 (5.8)	13
Medically unexplained	10.90 (5.5)	90	8.68 (5.3)	35	12.31 (5.2)	55

Bonferroni correction.

^a^Significantly different from other bone pathology, arthritis and PID/backache.

^b^Significantly different from sprain and others and wound.

^c^Significantly different from wound.

PID = pelvic inflammatory disease.

### Statistical analysis

All data were entered and analysed on SPSS version 14.0 (SPSS, Chicago, IL, USA). In order to assess the variables that were significantly associated with SRQ scores we used analysis of variance (ANOVA) for ordinal variables and Pearson's correlations coefficient for continuous variables as SRQ scores were normally distributed and homogeneity of their variance was confirmed. Variables found to be significantly associated with SRQ score were then added as covariates in an analysis of covariance (ANCOVA), which examined the relationship between orthopaedic diagnosis and SRQ score. *Post hoc *(Bonferroni) tests were subsequently performed to identify which particular orthopaedic diagnoses were associated with higher SRQ scores. Linear regression analysis was used to examine the association between total SRQ score (dependent variable) and all the sociodemographic variables, pain, disability score and orthopaedic diagnostic groups as independent variables.

## Results

Data were collected from 978 participants (response rate of 90.5%) 408 male and 570 female. Table 2 shows that the mean SRQ score was higher for females, participants who were divorced, separated or widowed, for those with little or no education, and with a low income. Significant positive associations were also found between SRQ total score and age (r = 0.16, *P *< 0.001), number of children (r = 0.18, *P *< 0.001) and the three social support scale scores indicating low support: item 1 (r = -0.30, *P *< 0.001), item 2 (r = -0.23, *P *< 0.001) and item 3 (r = -0.25, *P *< 0.001). After controlling for age and sex, SRQ score was associated with disability score (r = -0.098, *P *= 0.002) but not with pain severity (r = 0.05, *P *= 0.12). There was no difference in SRQ score between patients with medically explained (by trauma or organic disease) or unexplained symptoms (Table 2).

**Table 2:** Mean (standard deviation) Self-Rating Questionnaire (SRQ) total scores by demographic variables

	Number	Mean (SD) SRQ total score	*P *value
Female	570	12.50 (5.4)	*P *< 0.001
Male	408	8.45 (5.2)	
No formal education	530	12.01 (5.6)	*P *< 0.001
Primary school only	122	10.16 (5.8)	
Secondary school only	174	9.61 (5.7)	
Beyond secondary school	129	8.08 (4.9)	
Single	190	8.96 (5.6)	*P *< 0.001
Married	719	11.04 (5.6)	
Divorced/separated/widowed	64	13.84 (5.2)	
Income < Rs 4,000	647	11.69 (5.6)	*P *< 0.001
Rs 4,000 to 6,000	168	9.35 (5.6)	
Rs 6,001 to 8,000	71	8.20 (5.4)	
>Rs 8,000	71	8.83 (5.3)	
Medically explained	887	10.81 (5.8)	*P *= 0.79
Medically unexplained	91	10.89 (5.5)	

An SRQ score of 9 or more, which indicates probable depressive disorder, occurred in 46.8% of men and 76.0% of women. Among men with medically unexplained symptoms 19/35 (54.3%) had an SRQ score of 9 or more compared with 172/373 (46.1%) of those with symptoms explained by organic pathology (χ^2 ^= 0.868; *P *= 0.35). Corresponding figures for women were 42/55 (76.4%) and 391/515 (75.9%) (χ^2 ^= 0.00; *P *= 0.92). Overall, 233/978 (23.8%) of our sample reported that they had entertained ideas of ending their life: 219/624 (35.1%) of those who scored more than 9 on SRQ and 14/354 (4.0%) of the remainder.

There were significant differences in total SRQ score between the diagnoses made by the orthopaedic surgeon (F = 4.59, *P *< 0.001) (Table 1). The patients with a diagnosis of other bone pathology, arthritis, or backache had the highest scores and Bonferroni comparisons revealed that these scores were significantly higher than the 'major fracture' group. Using ANCOVA that controlled for the effects of the participants' gender, age, marital status, level of education, number of children and monthly income, the significant difference by diagnostic group remained (*P *= 0.016) (Table 3).

**Table 3:** Mean (standard error) of the mean Self-Rating Questionnaire (SRQ) scores by orthopaedic diagnosis adjusted for age, sex, years of education, marital status, number of children and income

Diagnosis	Adjusted mean	Standard error of the mean
Major fracture	10.7	0.36
Small fracture	10.4	0.64
Other bone pathology	12.1	0.61
Sprain and others	8.5	1.07
Arthritis	10.9	0.36
PID/backache	12.2	0.67
Dislocation	9.1	1.65
Frozen joint	9.7	1.27
Wound	10.1	0.51
Other	8.6	1.17
Medically unexplained	11.1	0.57

PID = pelvic inflammatory disease.

In linear regression analysis when we controlled for all sociodemographic characteristics, disability and pain, arthritis, backache/prolapsed intervertebral disc, other bone pathology and major fracture continued to be associated with a raised SRQ scores indicating greater psychological distress (Table 4). Medically unexplained symptoms were associated with lower SRQ score but this failed to reach significance.

**Table 4:** Multiple regression to predict Self-Rating Questionnaire (SRQ) score

	Unstandardised coefficients		*P *value	95% confidence interval for B	
			
	B	Standard error		Lower bound	Upper bound
Age	0.004	0.016	0.82	-0.027	0.034
**Sex**	**2.687**	**0.388**	**<0.000**	**1.926**	**3.448**
Single	0.080	0.560	0.88	-1.018	1.179
Widowed/divorced/separated	1.370	0.779	0.079	-0.159	2.898
**Income**	**-0.482**	**0.199**	**0.015**	**-0.872**	**-0.093**
Number of children	0.132	0.075	0.080	-0.016	-0.280
**Social support**	**-0.525**	**0.064**	**<0.000**	**-0.652**	**-0.399**
Education	-0.279	0.171	0.103	-0.614	0.057
**BDQ score**	**-0.223**	**0.075**	**0.003**	**-0.370**	**-0.075**
**VAS pain**	**0.025**	**0.008**	**0.001**	**0.010**	**0.040**
**Arthritis**	**1.554**	**0.693**	**0.025**	**0.193**	**2.915**
**PID/backache**	**2.844**	**0.877**	**0.001**	**1.122**	**4.566**
**Major fracture**	**1.662**	**0.690**	**0.016**	**0.309**	**3.016**
Small fracture	1.049	0.867	0.227	-0.652	2.749
**Other bone pathology**	**2.660**	**0.851**	**0.002**	**0.989**	**4.331**
Wound	0.606	0.769	0.431	-0.904	2.116
Medically unexplained symptoms	0.013	0.008	0.097	-0.002	0.029

Bold type indicates significance.

PID = pelvic inflammatory disease; VAS = visual analogue scale.

## Discussion

This is, we believe, the first study from a low-income country concerning depression in a large sample of orthopaedic outpatients. The prevalence of depression in our sample was high with an SRQ score of 9 or more, which indicates probable depressive disorder, occurring in 45.6% of men and 76.1% of women. These proportions are almost identical to those we recorded in a similar consecutive sample of patients attending medical clinics at the same hospital (47% of men and 63% of women) [18] and are much higher than those we previously found in a population-based sample in rural Pakistan (17.5% of men and 44.1% of women) [24]. In the latter study all but one of the people who scored nine or more on the SRQ had depressive disorder ascertained by research interview. Thus this cut-off point appears to indicate probable depressive disorder rather than emotional distress.

Our results are consistent with studies from the West, which have reported that the prevalence of psychological disorder following physical trauma in a variety of settings is 23% to 41% [9,11,12,15,25]. Such proportions are much higher than those found in the general population and somewhat higher than those reported in some medical clinics in the West [12]. As a result of methodological differences our results cannot be directly compared with those reported from the west but it is notable that in both USA and Pakistan trauma patients have a very high prevalence of probable depressive disorder.

Our primary hypothesis that the prevalence of depression would be higher in patients with medically unexplained musculoskeletal symptoms was disproved. Although patients with medically unexplained musculoskeletal pains experienced great psychological distress the level was even higher in patients with arthritis, backache and other bone pathology. This is different from studies in high-income countries [26], and our own previous study in medical clinics in Pakistan [18], which indicated that depression is often associated with 'medically unexplained symptoms'. The patients included in this study are clearly very distressed with a high proportion entertaining suicidal ideas: 24% compared to 5% in our previous population study [16]. Thus we anticipate that many of these patients need treatment for depression.

It is apparent that the usual sociodemographic factors are clearly associated with a high SRQ score (female, little education, financial difficulties, lack of social support, divorced, separated or widowed status, numerous children) [16]. It is probable that many of these people may have been depressed before their fracture or illness because of their life circumstances. It is impressive, therefore, that several orthopaedic diagnoses added additional variance to the explanation of SRQ score in multiple regression. This indicates that the presence of such illness is associated with even greater distress than can be accounted for by sociodemographic circumstances. This may be understandable in terms of the further hardship such illness brings in a setting of poverty and hardship. These orthopaedic diagnoses will probably have prevented these patients from earning a living or doing their usual household and other tasks.

The main limitation of this study is the fact that we did not perform second stage interviews to ascertain definite psychiatric diagnoses. It is possible that physical symptoms could have inflated the SRQ score but we controlled for severity of pain and level of physical disability in the multiple regression analysis and still we found that the diagnoses of arthritis, other bone pathology, backache/pelvic inflammatory disease (PID) and major fracture were significantly associated with increased SRQ score. Our previous work indicates that the cut-off of 8/9 is the most appropriate to detect psychiatric disorder in primary care clinics in Pakistan. The high proportion with suicidal ideas testifies to the very high level of distress, and probable depressive disorder experienced by many of these patients. A further limitation of only using the SRQ as a measure of depression is the gender differences in the psychometric properties of the SRQ. In our earlier work a cut-off score of 5/6 was found to be better for males and, had we used this cut-off in the present study the prevalence figure for men would be higher. Since our main findings in this study are concerned with the total SRQ score, the cut-off is not affecting our main results concerned with the relationship between total SRQ and correlated features. We used the conventional cut-off so that our prevalence figures are consistent with all the previous studies from Pakistan, which have used the same cut-off scores for both men and women.

The main strength of the present study is the high response rate and we believe that our sample is not biased towards those patients who might have been prepared to undergo a more detailed research interview at the clinic visit, which would have been required to establish psychiatric diagnosis with certainty. As this is a cross-sectional study we cannot establish for certainty the level of distress that these patients experienced before their current illness, although we know the level of depression in the population is high [17]. It is possible that they were depressed prior to their injury and actually experienced injury as a result of their depression. We were not made aware of any participants who had actually injured themselves deliberately; accidents are more common while someone is depressed and preoccupied. It is very likely that the injury and illnesses exacerbated pre-existing depressive illness.

## Conclusions

Depressive disorder appears to be very common in orthopaedic outpatients in Pakistan. Orthopaedic surgeons may need assistance to develop methods of screening for depression either by using a questionnaire, such as SRQ, or by asking pertinent questions. Patients who screen positive can be treated immediately with antidepressants and/or referred for further assessment and treatment by the mental health professionals. Treatment of depression in this population should aid complete recovery, though prospective and treatment studies are required to demonstrate this. We need to develop ways of achieving this in a manner that is compatible with the brief time available to doctors in outpatient clinics in Pakistan. This study demonstrates again the massive unmet mental health need in developing countries [3,27,28] and that novel forms of treatment are required.

## Competing interests

The authors declare that they have no competing interests.

## Authors' contributions

All authors contributed to the conception and design of study. NH, SMH, IBC, HR and FC wrote the protocol. SMH and RR collected the data. NH, IBC, HR, RR and FC undertook the statistical analysis and NH wrote the first draft of the manuscript. All authors contributed to and approved the final manuscript.
